# Seasonal Variation in Generic and Disease-Specific Health-Related Quality of Life in Rhinologic Patients in Southern Finland

**DOI:** 10.3390/ijerph18126428

**Published:** 2021-06-14

**Authors:** Maija Ylivuori, Reija Ruuhela, Harri Sintonen, Paula Virkkula, Risto P. Roine, Maija Hytönen

**Affiliations:** 1 Department of Otorhinolaryngology—Head & Neck Surgery, Helsinki University Hospital, Kasarmikatu 11–13, 00029 Helsinki, Finland; paula.virkkula@hus.fi (P.V.); maija.hytonen@hus.fi (M.H.); 2 Weather and Climate Change Impact Research, Finnish Meteorological Institute, 00560 Helsinki, Finland; reija.ruuhela@fmi.fi; 3 Department of Public Health, University of Helsinki, 00014 Helsinki, Finland; harri.sintonen@helsinki.fi; 4 Group Administration, University of Helsinki and Helsinki University Hospital, 00100 Helsinki, Finland; risto.p.roine@gmail.com; 5 Department of Health and Social Management, University of Eastern Finland, 70211 Kuopio, Finland

**Keywords:** adult, climate, PROM, sinonasal, questionnaire, 15D, SNOT-22

## Abstract

Background: Seasonal variation in exacerbations, hospitalisations, and mortality statistics has been reported for some diseases. To our knowledge, however, no published studies exist on the seasonality of health-related quality of life (HRQoL) amongst rhinologic patients. Aims/Objectives: This study, therefore, aimed to investigate the possible seasonal variation in rhinologic patients’ HRQoL using the rhinologic disease-specific Sino-Nasal Outcome Test-22 (SNOT-22) and the generic 15D HRQoL instrument. Material and Methods: We enrolled unselected adult rhinologic patients requiring specialist care at the Helsinki University Hospital in this cross-sectional, questionnaire-based prospective study during four seasons: February (winter), May (spring), August (summer), and November (autumn). Patients received SNOT-22 and 15D questionnaires via post. The Finnish Meteorological Institute supplied climate data from these months. Results: SNOT-22 and 15D data were available for 301 and 298 patients, respectively. We found no statistically significant differences (*p* = 0.948) between the mean monthly 15D scores or mean SNOT-22 scales. Furthermore, the mean SNOT-22 subscales did not differ between the monthly study periods. Conclusions and Significance: Our study shows that seasonality did not impact rhinologic patients’ SNOT-22 or 15D HRQoL scores. Thus, these questionnaires can be used for follow-up amongst rhinologic patients regardless of season.

## 1. Introduction

Some medical conditions vary by season. For instance, changes in the outdoor temperature, humidity, solar radiation, weather type, and air pollution can influence patients’ symptoms and disease severity. The climate, and changes to it, impact individuals’ lives both locally and globally. Temperature, humidity, air pollution, and many other factors affect morbidity, disease exacerbation, and even mortality. Due to global climate change, research on how climate affects health has become increasingly important. Yet, given that we identified only a few studies on this subject, little is known about the seasonality of health-related quality of life (HRQoL). 

Earlier studies have reported a seasonal variation in hospital admissions for some abdominal infections. For example, the incidence of acute appendicitis decreases significantly during the cold months of the year in Finland [1]. Another study in the United States showed that general surgery-related emergency hospitalisations follow a consistent cyclical pattern, with more admissions occurring during the summer months [2]. While no correlation was detected between temperature and the incidence of non-specific abdominal pain, humidity significantly impacted pain [1]. Seasonal variation has also been found in several rheumatic diseases [3], blood pressure [4], and in obstructive sleep apnoea [5]. Amongst the elderly, seasons affect mortality, medical care expenditures, and institutionalisation, all of which are highest during winter [6]. Furthermore, seasonal incidence and mortality due to sepsis appear highest during winter [7]. In addition, globally, the rate of suicides and attempted suicides peak during spring [8].

Seasonal variation has been observed in upper respiratory tract infections [9]. Asthma exacerbations, as well as asthma-related hospitalisations and mortality in adults, are highest during winter and spring [10]. Moreover, cold spells are associated with increased mortality rates in populations around the world, and a body of evidence suggests that cold spells also carry other adverse health effects [11]. Seasonal variation in symptoms commonly occurs in patients with allergic diseases. In addition, epistaxis more frequently occurs during winter [12]. Incidental sinus changes appearing on MRI represent common findings, and appear to be seasonally influenced. Furthermore, more sinus abnormalities occur in winter, although they bear little association with symptoms [13].

Only a few studies have examined the seasonality of generic health-related quality of life. For instance, Jia et al. (2009) found that physical HRQoL amongst the US population was best during summer and worst during winter; mental health, by contrast, is worst during spring and autumn. Thus, they encouraged researchers to note the time of year during which the data were collected when comparing data from different studies, since seasons can influence patients’ health [14]. Similarly, Grimaldi et al. (2008) observed that HRQoL in a Finnish population was influenced by both seasonal changes in mood and behaviour, as well as indoor illumination [15].

Further reports identified the following environmental aspects affecting rhinologic patients: indoor and outdoor temperature, relative and absolute air humidity, allergens, and air pollutants [16]. In a study amongst healthy subjects, individuals’ perceptions of nasal patency showed a significant increase in both cooler and drier air [17]. 

A recent article found that chronic rhinosinusitis (CRS) patients have seasonal variation in disease-specific HRQoL, and they have the most severe symptoms during the winter; this variation may be mainly explained by mood [18]. Based also on our own clinical experience, a common opinion amongst clinicians is that the symptoms and disease-specific HRQoL of rhinologic patients worsen during the colder and drier winter season, and improve during the warmer and more humid summer season. 

Yet, we found no published studies regarding the seasonal variation of generic or disease-specific HRQoL amongst other rhinologic disease patients. Thus, this study aimed to assess the possible seasonal variation of both disease-specific and general HRQoL amongst patients experiencing different diseases in the nose or sinuses.

## 2. Patients and Methods 

We designed a cross-sectional, questionnaire-based study to collect information on HRQoL amongst patients with different rhinologic diseases. We invited all adult (≥18 years) patients referred to the Helsinki University Hospital Department of Otorhinolaryngology—Head and Neck Surgery due to rhinologic disease or symptoms within the study months in 2014 to participate. A medical history questionnaire, a generic HRQoL questionnaire (15D), and the rhinologic disease-specific Sino-Nasal Outcome Test-22 (SNOT-22) HRQoL questionnaire were mailed via post to enrolled patients. We asked patients to complete the surveys within three days and return them to us, and that all patients provide their written informed consent. For this study, the study periods consisted of the following. February: 1 February to 15 March, May: 1 May to 15 June, August: 1 August to 15 September, and November: 1 November to 15 December. We also collected data for a small pilot study in August and November 2013. Finally, the Finnish Meteorological Institute provided climate data. 

The generic 15D questionnaire is a standardised, validated, and self-administered HRQoL instrument that can be used both as a profile and a single-index measure (http://www.15d-instrument.net/15d/, accessed on 22 November 2018), consisting of the following 15 dimensions: moving, seeing, hearing, breathing, sleeping, eating, speech, excretion, normal activities, mental functioning, discomfort and symptoms, depression, distress, vitality, and sexual activity. For each dimension, the respondent must choose one of the five levels best describing her/his health status at that moment (best level = 1; poorest level = 5). Missing answers (provided there are no more than three) along the 15D dimensions can be predicted using linear regression with age, gender, and answers to the other dimensions serving as independent variables. The valuation of the 15D relies on the application of the multi-attribute utility theory. A set of utility or preference weights elicited from the general public through a three-stage valuation procedure provides an additive aggregation formula to generate the utility score—that is, the 15D score (single-index number) across all dimensions. The maximum score is 1 (no problems on any dimension) and the minimum score is 0 (equivalent to being dead). The minimal clinically important difference (MID) in the 15D score is ±0.015. Overall, the 15D is a sensitive instrument capable of detecting cross-sectional differences and changes over time in HRQoL. 

SNOT-22 is a widely used rhinologic HRQoL instrument. We know the average SNOT-22 scores among the general population, and well-defined guidelines for analysis of SNOT-22 scores exist. More specifically, the minimal clinically important difference in SNOT-22 has been reported as equaling 8.9. When comparing 15 different disease-specific sinonasal outcome tests for chronic rhinosinusitis (CRS), SNOT-22 was identified as most suitable. Whilst SNOT-22 was originally developed for CRS assessment, it serves as a useful tool in other clinical situations, such as in nasal septal surgery and in septorhinoplasty. As a tool, SNOT-22 consists of 22 questions (items) related to sinonasal symptoms and quality of life. In the questionnaire, patients rate each item from 0 to 5 (0 = no problem, 1 = very mild problem, 2 = mild or slight problem, 3 = moderate problem, 4 = severe problem, or 5 = problem as bad as it can be). The total maximum score for SNOT-22 reaches 110 points (22 × 5). If the patient chooses more than one option for a question (for example, 1 and 2), the mean (1.5) is recommended for analysis (Claire Hopkins, personal communication). Missing answers are dealt with as follows: if patients completed more than 50% of the SNOT-22 questionnaire, missing items are imputed from the mean of completed items. When more than 50% of the SNOT-22 items are missing, the patient is excluded from the analysis. 

For analysis, the SNOT-22 questionnaire can also be divided into subscales [19]. In this study, we used the following subgroups, originally published by Feng et al. (2017): nasal symptoms (questions 1–8), otologic/facial pain (questions 9–12), sleep (questions 13–20), and emotional subdomains (questions 21–22). The maximum scores for these subgroups are 40, 20, 40, and 10, respectively, depending on the number of items included in the subgroups. Some investigators suggest using the subgroup scores instead of the total SNOT-22 scores for more clinically meaningful results [20].

In our statistical analysis, we performed the analysis of variance when comparing the population means. When the requirement for a normal distribution was not met, we relied on the Kruskal–Wallis one-way ANOVA instead. The normality and equal variance assumptions were tested using the D’Agostine omnibus test of normality and Brown–Forsythe test of homogeneity, respectively. When appropriate, power transformations were applied before statistical testing to achieve normality of the data. We considered a difference between the groups as statistically significant at *p* < 0.05. The research ethics board of the Helsinki University Hospital approved the study protocol (Dnro 128/13/03/02/2013).

## 3. Results 

In total, 349 patients returned questionnaires. Three patients were under 18 years old and were therefore excluded from further analysis. In addition, 43 patients did not answer questionnaires within the defined study period months, and were excluded from the seasonal analysis. Furthermore, two SNOT-22 and five 15D patients returned incomplete questionnaires, and were excluded from further analysis. Our study, thus, consisted of SNOT-22 data from 301 (mean age = 50.5 years; 48.8% men) patients and 15D data from 298 (mean age = 50.5 years; 49% men) patients. The most common rhinologic diseases consisted of CRS (*n* = 105; 35%), followed by non-allergic rhinitis (*n* = 59; 20%), allergic rhinitis (*n* = 38; 13%), septal deviation (*n* = 30; 10%), anosmia/hyposmia (*n* = 9; 3.0%), and epistaxis (*n* = 8; 2.6%). We calculated the response rate during one month (November 2014), which reached 46.3%, finding no statistically significant age or gender differences between respondents and non-respondents (mean age = 48.6 and 44.1 years; 47.2% and 46.0% men, respectively).

**Figure 1:**
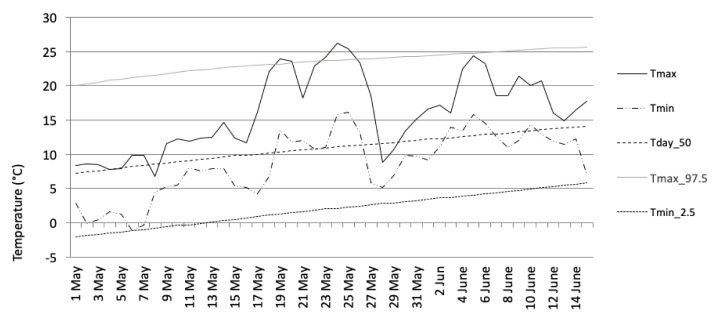
Daily maximum and minimum temperatures at Helsinki–Kaisaniemi during the second survey period (May). Tmax = maximum temperature, Tmin = minimum temperature, Tday_50 = 50th percentile of daily mean temperature distribution, Tmax_97.5 = threshold for exceptionally high temperatures (or the 97.5 percentile of the daily maximum temperature distribution), Tmin_2.5 = threshold for exceptionally low temperatures (or the 2.5 percentile of daily minimum temperature). The percentiles were determined for the normal climate period from 1981 to 2010.

In 2014, the monthly mean temperatures and mean absolute humidity recorded at the Helsinki–Kaisaniemi weather station were +0.2/4.7 (February), 10.6/8.7 (May), 17.9/11.9 (August), and 3.2 °C/5.5 g/m^3^ (November). The normal temperature values were −6, +10, +15, and 0 °C, respectively (Table 1). In addition, as an example of a monthly temperature variation, the May study period appears in Figure 1. The beginning of the survey period in May was unseasonably cold, but a warm period, which lasted for more than one week, began in the middle of May. Another shorter warm period took place at the beginning of June. 

**Figure 2:**
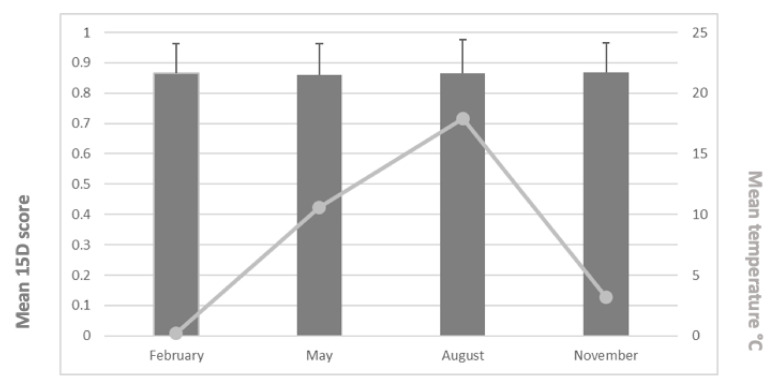
Mean 15D scores (and SD values) and mean temperatures for the study periods.

**Figure 3:**
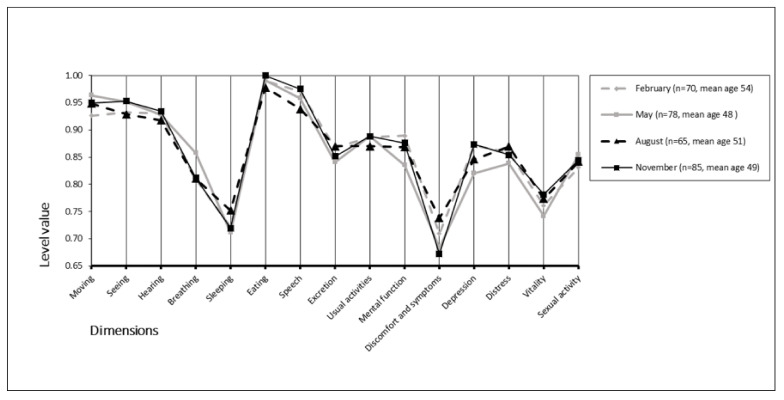
Seasonality of the 15D profiles.

The mean (±SD) 15D scores were 0.865 (0.096) in February, 0.860 (0.102) in May, 0.865 (0.114) in August, and 0.868 (0.097) in November. We found no statistically significant differences between groups (*p* = 0.968). The mean 15D scores and mean temperatures appear in Figure 2. Furthermore, we found no statistically significant differences across the 15D dimensions between the different monthly study periods (*p* = 0.149–0.936) (Figure 3). 

**Figure 4:**
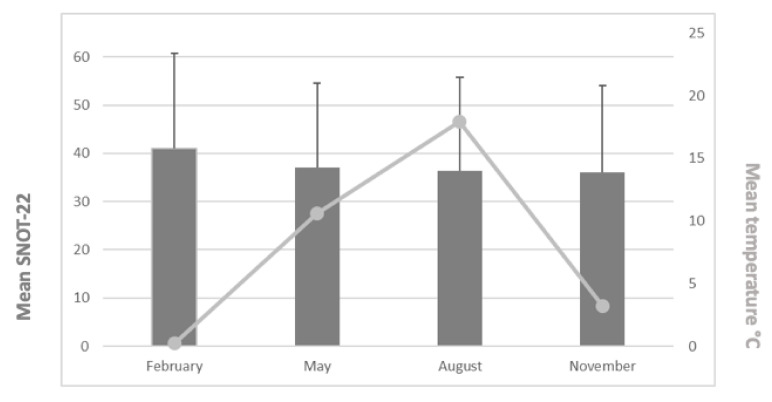
Mean SNOT-22 values (and SD values) and mean temperatures for the study periods.

Figure 4 provides the mean SNOT-22 values and mean temperatures during the study periods. The mean SNOT-22 values were 41.0 (February: *n* = 72, min = 0, max = 93, range = 93, standard deviation (±SD) = 19.8), 37.0 (May: *n* = 78, min = 1, max = 79, range = 78, SD = 17.5), 36.4 (August: *n* = 65, min = 2, max = 99, range = 97, SD = 19.3), and 36.0 (November: *n* = 86, min = 2.5, max = 76, range = 73.5, SD = 18.1). We found no statistically significant differences between the mean SNOT-22 values across the study months (*p* = 0.385). Patients’ age or gender did not have a statistically significant impact either.

**Figure 5:**
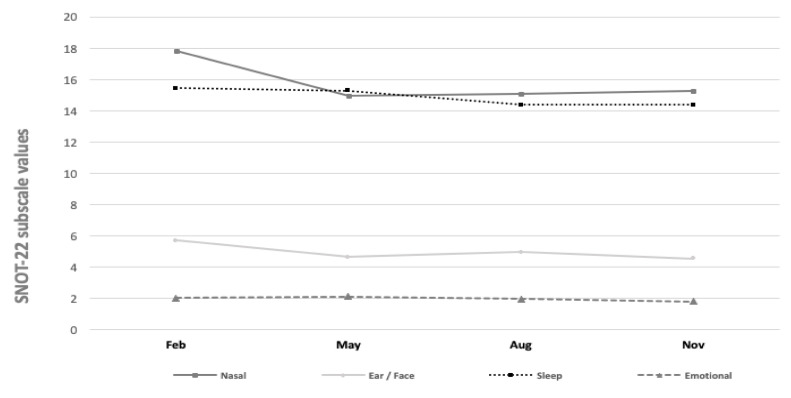
SNOT-22 subscale values during different month periods.

Finally, Figure 5 displays the SNOT-22 subscale values across the different study months. The mean values of the SNOT-22 question subscales for nasal symptoms varied between 14.96 and 17.81 (*p* = 0.14). The values of otologic/facial pain (4.54–5.71, *p* = 0.36), sleep (14.38–15.45, *p* = 0.84), and emotional subdomains (1.81–2.11, *p* = 0.77) varied. Yet, we found no statistically significant seasonal variations across the subscales. 

## 4. Discussion

In this cross-sectional, prospective, and questionnaire-based study, we investigated the possible seasonal variation in HRQoL amongst rhinologic patients. We found no seasonal variation in the generic 15D scores, nor the total disease-specific SNOT-22 HRQoL scores in patients with various rhinologic diseases. Furthermore, we observed no statistically significant seasonal impact when the SNOT-22 questionnaire was divided into subscales. To our knowledge, this represents the first study in which the possible effect of seasonal weather variation associated with general and disease-specific HRQoL in groups of unselected rhinologic patients was investigated.

In some medical conditions, for example, in rheumatic diseases, obstructive sleep apnoea, upper respiratory infections, and asthma, earlier studies have reported seasonal variation [3,4,5,9,10]. Talat et al. evaluated the seasonal variation in disease-specific HRQoL of CRS patients. According to their results, CRS patients’ SNOT-22 scores have statistically but not clinically significant seasonal variation, which is caused by the higher scores in the sleep and emotion subdomains in winter [18]. This might be explained by the higher prevalence of depression in CRS patients compared to healthy controls [21]. We, however, did not find any seasonal variation amongst rhinologic patients. The reason could be that, in our study, we used HRQoL measurements that extensively, in many ways, measures the burden of the illness. In most earlier published studies, the measurements focus mainly on the symptoms or findings of the disease.

HRQoL instruments are used in clinical practice and trials as tools to provide evidence, for example, of disease severity and treatment benefit from the patient perspective [22]. According to our results, that seasonal variation has no statistical or clinically significant effect on rhinologic patients’ quality of life. This will facilitate future research setups, as the season does not need to be considered.

The strength of the study lies in its geographic location, since Finland is situated between approximately 60° and 70° latitudes. It is a country with four distinct seasons, and, therefore, it experiences warm summers and cold winters. Furthermore, the number of patients was similar during each study period, with a nearly equal gender distribution, and we did not find statistically significant differences between gender or age. Moreover, we found no statistically significant age or gender differences between respondents and non-respondents in this study. One limitation of this study relates to the surveys, whereby they were conducted only during one year. Consequently, we cannot compare the possible inter-annual variability of the general and disease-specific QoL measurements and the impact of varying weather conditions on these outcomes. A further possible limitation is that the mean temperatures during the study periods appeared somewhat different from the normal climatic values. The largest differences were recorded for February (+0 °C vs. −6 °C); this resulted in reduced exposure to cold thermal conditions compared to more typical winter conditions. However, the mean 15D and SNOT-22 scores were quite stable, and clinically important differences were not achieved. Perennial allergic rhinitis affects HRQoL [23], and seasonal allergic rhinitis may decrease HRQoL during pollen season [24]. We had, however, very few patients suffering from seasonal allergy (*n* = 7, 2.3%) in our data.

## 5. Conclusions

To conclude, our study demonstrates that there is no statistically significant seasonal variation in generic or disease-specific HRQoL amongst patients with different rhinologic diseases or symptoms when analysed in a country with four distinct seasons. This establishes the use of the 15D and SNOT-22 as relevant tools in clinical practice and research, since considering the role of seasons does not seem necessary.

## Figures and Tables

**Table 1:** Climate statistics taken from the Helsinki–Kaisaniemi weather station during the survey periods in 2014. Tmean = mean temperature, ΔTmean = deviation from the mean temperature from the normal climate (1981–2010), Tmax_avg = average daily maximum temperature, ΔTmax = deviation from the maximum temperature from normal, Tmin = average daily minimum temperature, ΔTmin = deviation from the minimum temperature from normal, Rel Hum = relative humidity, Abs Hum = absolute humidity.

	Tmean (°C)	ΔTmean (°C)	Tmax_avg (°C)	ΔTmax (°C)	Tmin_avg (°C)	ΔTmin (°C)	Rel Hum—mean (%)	Abs Hum—mean (g/m^3^)
1 Feb–15 Mar	1.0	+4.9	2.7	+3.9	−0.5	+6.2	90	4.7
1 May–15 Jun	12.1	+0.8	16.2	+0.7	8.6	+1.1	79	8.7
1 Aug–15 Sept	16.9	+1.7	20.7	+2.2	13.6	+1.6	83	11.9
1 Nov–15 Dec	3.0	+2.2	4.5	+1.6	1.5	+2.9	91	5.5

## Data Availability

The data presented in this study are available on request from the corresponding author. The data are not publicly available due to privacy.
